# Age‐associated changes of resting energy expenditure, body composition and fat distribution in Chinese Han males

**DOI:** 10.14814/phy2.13940

**Published:** 2018-12-08

**Authors:** Nousayhah Amdanee, Wenjuan Di, Juan Liu, Jing Yu, Yunlu Sheng, Shan Lv, Mohammad Ridwan Chattun, Hanmei Qi, Wangyan Liu, Lijun Tang, Guoxian Ding

**Affiliations:** ^1^ Department of Geriatrics The First Affiliated Hospital of Nanjing Medical University Nanjing China; ^2^ Department of Psychiatry The Affiliated Nanjing Brain Hospital of Nanjing Medical University Nanjing China; ^3^ Department of Radiology The First Affiliated Hospital of Nanjing Medical University Nanjing China

**Keywords:** Fat free mass, fat mass, resting energy expenditure, subcutaneous fat, visceral fat

## Abstract

Age‐related alterations in whole body composition, particularly, reduced fat free mass (FFM) and increased fat mass (FM), lead to a progressive decline in resting energy expenditure (REE). Similarly, regional body composition and fat distribution changes with age might also contribute to an overall lower REE. This study investigated the influence of age on REE, regional body composition and fat distribution, including subcutaneous fat (SF) and visceral fat (VF), in a Chinese Han population as well as their contributions to age‐related changes in REE. One hundred and two males aged 31–83 years old underwent dual‐energy X‐ray absorptiometry (DXA) which measured whole body and regional FM and FFM. SF and VF were measured by magnetic resonance imaging (MRI) and REE by indirect calorimetry. Age was significantly negatively correlated with REE (*r = *−0.37), total FFM (*r = *−0.25), upper limbs FFM (*r = *−0.32), lower limbs FFM (*r = *−0.34) and showed positive association with trunk FFM (*β*=0.926). FM, SF and VF decreased in older age groups after an initial rise up to 55–65 years. REE correlated positively to FM, FFM, SF, VF and showed significant association with age (*β* = −0.254) independent of age‐associated changes in body composition. The regional alterations in body composition with age were explained by changes in trunk FFM (*β* = 0.926). Age‐related decline in REE were not solely due to alterations in FM and FFM. Therefore, the changes in regional body composition, fat distribution and REE which occur during aging could be explained by disparities in race, ethnicity, diet, physical activity, and lower specific metabolic rates of FFM components.

## Introduction

Alterations in body composition, which occur during aging, have been linked to increased disability, morbidity, and mortality in the geriatric population (Goya Wannamethee et al. 2004; Schaap et al. 2013). These changes are caused by an impairment in energy balance, whereby weight gain and weight loss are induced by a positive energy balance and negative energy balance, respectively (Roberts and Rosenberg 2006). A prolonged energy dysregulation could pave the way for advanced age‐related disorders such as obesity, malnutrition, hypertension, and diabetes (Poehlman 1993).

Resting energy expenditure (REE), a key component of energy balance regulation (Poehlman 1993), contributes to 50–70% of the total energy expenditure of human metabolism (Elia et al. 2000). There is a progressive decline in REE during aging due to a reduction in fat free mass (FFM) and an increase in fat mass (FM) (Hunter et al. 2001; Manini 2010; Geisler et al. 2016). FFM has a REE variability of 43–85% among individuals (Poehlman 1993; Illner et al. 2000; Bosy‐Westphal et al. 2003). The high metabolic components of FFM namely, the visceral organs and the brain, account for 70–80% of REE while the low metabolic components including muscle mass and skeletal bone provide for 20% of REE (Illner et al. 2000; Wang et al. 2000; Müller et al. 2013). A decrease in FFM and the metabolic activity of its components were predominantly associated with an age‐related reduction in REE (Illner et al. 2000). Wang et al. (2010) reported a significant decline in the specific resting metabolic rates of major organs and tissues in elderly individuals. In contrast, the less metabolically active FM, which also influences REE, is increased in the elderly (Poehlman 1993; Illner et al. 2000). With age, an increase in abdominal adiposity posed a higher mortality risk in comparison to total body adiposity (Kuk et al. 2009).

Abdominal FM, the main site of fat redistribution and accumulation in the human body, is a precursor to metabolic and cardiovascular diseases (Zhang et al. 2008). There is a greater proportion of visceral fat (VF) compared to subcutaneous fat (SF) in the abdomen. Both visceral and SF increase with age, but only VF increases irrespective of gender and ethnicity (Kuk et al. 2009). In a study on the relationship between REE and fat distribution throughout the body, a significantly lower REE was associated with an increased abdominal VF (Jia et al. 2005). There was a negative association between REE and intra‐abdominal adiposity in the elderly (Hunter et al. 2001). However, REE was positively correlated to a decrease in total FFM, trunk FFM, appendicular FFM as well as lower limbs FM (Hunter et al. 2001). The Florey Adelaide Male Aging Study reported that aging was related to an increase in VF as well as total body and abdominal percentage FM. The rise in abdominal percentage FM and total body percentage FM were caused by a reduced lean mass and deposition of FM in the abdominal area, respectively (Atlantis et al. 2008).

It is crucial to examine the relationship of age‐related changes in REE with body composition (regional and whole‐body FM and FFM) and fat distribution in order to understand the mechanism of age‐associated modifiable conditions and hence, devise better approaches to prevent and manage the adverse health concerns associated with aging. Since a progressive increase in FM and concomitant loss of FFM with aging contributed to changes in REE, we hypothesized that age‐related changes in regional body composition and fat distribution could additionally influence REE measurements. Therefore, the purpose of this investigation was to examine the changes of regional body composition and fat distribution with aging and confirm whether the results from prior studies could be replicated in an aging Chinese Han males’ population in the presence of ethnical and cultural disparities. Another purpose of this study was to measure the contribution of whole body and regional FM and FFM, as well as SF and VF to age‐related changes in REE.

## Materials and Methods

### Subjects

One hundred and two healthy Chinese Han male adults, aged between 31 and 83 years old, were recruited during a general health check‐up in the outpatient department of The First Affiliated Hospital of Nanjing Medical University between 2013 and 2015. In order to assess the participants’ health status, resting blood pressure, heart rate, blood glucose, lipid profile, electrocardiogram, and chest X‐ray, were performed. All subjects had a normal physical examination, laboratory values and auxiliary examination. Subjects with unstable weight, defined as weight gain or weight loss >2.0 kg in the past 6 months, which could alter body composition were excluded. None of the subjects had a history of recent illness or have been hospitalized in the past 6 months. Individuals with cancer or chronic illnesses such as pulmonic, hepatic, renal, cardiovascular diseases which could affect body composition, were also ruled out.

### Anthropometric assessment

Body weight was measured to the nearest 0.1 kg using standard digital scales while subjects wore light clothing with no shoes. Body height (without shoes) was recorded to the nearest 0.1 cm by a digital ultrasound instrument. Waist and hip circumferences were measured by a Gulick tension tape to the nearest 0.1 cm in the horizontal plane. While the subjects had their weight evenly distributed on both feet, waist circumference was measured between the lower rib margin and the iliac crest. Hip circumference was recorded at the level of the largest lateral extension of the hips. Each measurement was done twice and the average obtained was used for further analysis.

### Dual‐energy X‐ray absorptiometry

Whole body and regional FM and FFM (appendicular, trunk, android and gynoid) were obtained with a dual‐energy X‐ray absorptiometry (Hologic Discovery A; Hologic Inc., Bedford, MA, USA) and analyzed by the Encore Software 11. All subjects were required to remove any metal objects or jewelry and lie supinely in the scanner. The upper and lower limbs regions were separated from the trunk by the glenohumeral joint and a line passing obliquely through the hip joint at 45°, respectively. The trunk area was estimated between the first cervical vertebra and femoral neck. Android area was taken from the pelvis cut to the upper boundary above the pelvis cut which is 20% of the distance between the pelvis and neck cuts. The gynoid area was defined from the lower boundary of the umbilicus to a line equal to twice the height of android area. Body fat percentage was obtained by dividing FM with total body mass.

### Magnetic resonance imaging

All participants underwent abdominal MRI using a multi‐slice 3.0 T MRI system (MAGNETOM trio, Siemens, Germany) with a phased‐array surface coil. The subcutaneous and visceral adipose tissue were estimated from T1‐weighted sequences at the lumbar level L4.

### Resting energy expenditure

REE was measured by an open‐circuit indirect calorimetry system (Quark PFT Ergo, COSMED SRL, Rome, Italy) and was analyzed by a canopy dilution technique. After a 12‐h fast, each participant was asked to breathe freely in a canopy hood which covered their head for 30 min. Before each measurement, the flowmeter was calibrated with a 3L syringe and a digital turbine flowmeter directly measured the flow rate. The continuously measured oxygen consumption and carbon dioxide production were converted by the Weir formula to determine REE.

### Statistical analyses

Residual mass (RM), the high metabolic component of FFM, was calculated as the difference between total body weight and the sum of adipose tissue, skeletal muscle mass, and bone mineral content. Pearson's correlation analyzed the relationship between the variables and linear regression analysis assessed the strength of relationship using SPSS 19.0 (SPSS Inc., Chicago, IL, USA). Multiple regression analyses were conducted to study the determinants of REE and uncover the body components which were significantly altered with age. All tests were two‐tailed and the significance level for all tests was set at *P *<* *0.05. To demonstrate the effect of age on mean REE, mean FM, mean FFM, mean fat percentage, mean SF, and mean VF, line charts were constructed. Line graphs showed the relationship of age with FM, FFM, and REE as well as the association of REE with FM and FFM.

## Results

The descriptive characteristics for the study population are shown in Table 1.

**Table 1:** Demographic characteristics of subjects

	All adults (*n = *102)
Age (years)	55 ± 10.14
Weight (kg)	76 ± 10.87
BMI (kg/m^2^)	25 ± 3.08
Waist circumference (cm)	93 ± 8.97
Hip circumference (cm)	99 ± 6.51
Subcutaneous fat area at L4 (cm^2^)	186 ± 58.14
Visceral fat area at L4 (cm^2^)	106 ± 37.89
Fat percentage (%)	74 ± 25.98
Total body FM (g)	20,188 ± 4724.67
FM of arms (g)	2505 ± 723.87
FM of legs (g)	5079 ± 1240.09
FM of trunk (g)	11,293 ± 3066.34
Android FM (g)	1970 ± 608.37
Gynoid FM (g)	2810 ± 651.47
Total body FFM (g)	54,548 ± 6922.81
FFM of arms (g)	6304 ± 1024.83
FFM of legs (g)	16,907 ± 2284.14
FFM of trunk (g)	27,001 ± 2805.49
Android FFM (g)	3965 ± 672.95
Gynoid FFM (g)	7908 ± 1092.79
Resting energy expenditure (kcal/day)	1784 ± 284.93
Residual mass (g)	23,766 ± 3339.43

Data are presented as mean ± standard deviation. BMI, body mass index; FFM, fat free mass; FM, fat mass.

**Figure 1:**
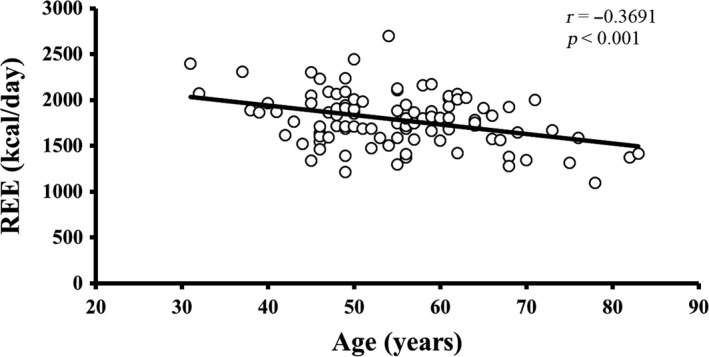
The relationship of REE with age. There is a decline in REE with advancing age. *r* is the Pearson's correlation coefficient and *P* *<* 0.05 indicates the significance level. REE, resting energy expenditure.

**Figure 2:**
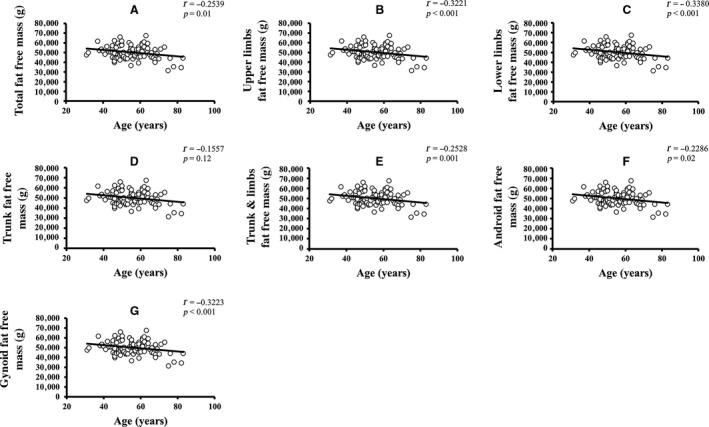
Age‐related changes in total body and regional FFM. (A–G) The linear relationship of different components of FFM changes with age (*P *<* *0.05 is denoted as the significant level and *r* represents Pearson's correlation coefficients). FFM, fat free mass.

**Figure 3:**
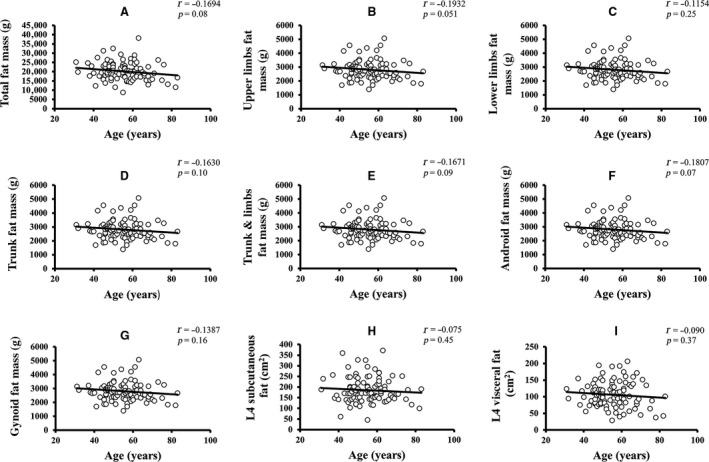
Age‐related changes in total body and regional FM. (A–I) The linear relationship of different components of FM changes with age (*P *<* *0.05 is denoted as the significant level and *r* represents Pearson's correlation coefficients). FM, free mass.

**Figure 4:**
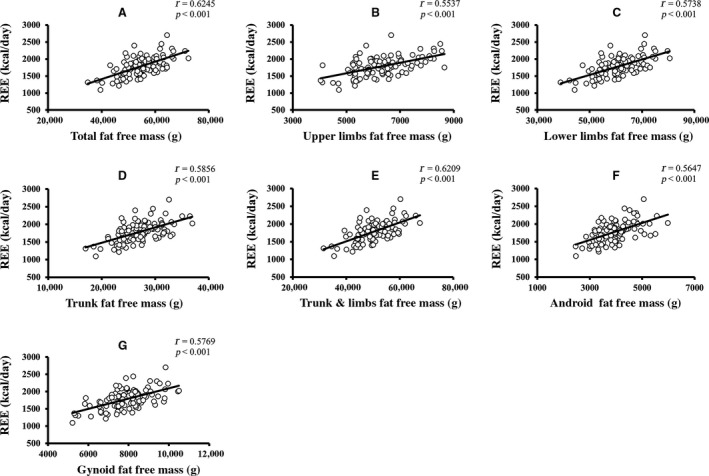
Relationship of REE with total body and regional FFM. (A–G) The linear relationship of different components of fat free mass with age‐related changes in resting energy expenditure (*P *<* *0.05 is denoted as the significant level and *r* represents Pearson's correlation coefficients).

**Figure 5:**
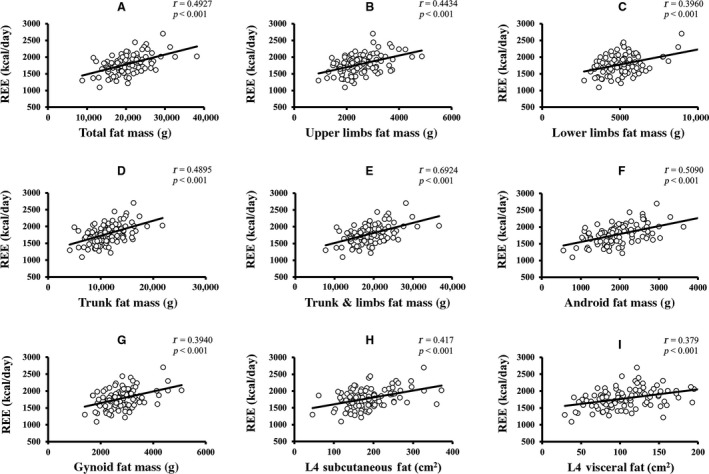
Relationship of REE with total body and regional FM. (A–I) The linear relationship of different components of FM with age‐related changes in resting energy expenditure (*P *<* *0.05 is denoted as the significant level and *r* represents Pearson's correlation coefficients). FM, free mass.

Pearson's correlation coefficients of age and REE with the parameters of body composition are summarized in Table 2. Age was significantly negatively correlated with REE, weight, body mass index (BMI), total FFM, upper limbs FFM, and lower limbs FFM. Age was more strongly correlated with FFM than with FM. None of the other variables showed a significant correlation with age. For age‐related changes in REE, see Figure 1. The linear relationship of age with regional and total body FFM is depicted in Figure 2. The age‐related changes in whole body and regional FM are shown in Figure 3. REE was significantly positively correlated with all the variables under investigation, including weight, BMI, total FM, percentage of body fat, appendicular FM, trunk FM, android FM, gynoid FM, total FFM, appendicular FFM, trunk FFM, SF, and VF. The REE‐related changes with total body and regional FFM and FM is displayed in Figures 4 and 5, respectively.

**Table 2:** Pearson's correlation coefficients of age and resting energy expenditure

	Age (years)		REE (kcal/day)	
	Pearson's coefficient	*P*‐value	Pearson's coefficient	*P*‐value
Weight (kg)	−0.2504	0.01^a^	0.5845	<0.001^a^
BMI (kg/m²)	−0.2449	0.01^a^	0.4825	<0.001^a^
FM (g)	−0.1694	0.08	0.4927	<0.001^a^
Upper limbs FM (g)	−0.1932	0.051	0.4434	<0.001^a^
Lower limbs FM (g)	−0.1154	0.25	0.3960	<0.001^a^
Trunk FM (g)	−0.1630	0.101	0.4795	<0.001^a^
Body Fat Percentage (%)	−0.0096	0.92	0.2464	0.01^a^
Android FM (g)	−0.1807	0.07	0.5090	<0.001^a^
Gynoid FM (g)	−0.1387	0.16	0.3940	<0.001^a^
Subcutaneous FM (g)	−0.67	0.50	0.417	<0.001^a^
Visceral FM (g)	−0.80	0.43	0.380	<0.001^a^
FFM (g)	−0.2539	0.01^a^	0.6245	<0.001^a^
Upper limbs FFM (g)	−0.3221	<0.001^a^	0.5537	<0.001^a^
Lower limbs FFM (g)	−0.3380	<0.001^a^	0.5738	<0.001^a^
Trunk FFM (g)	−0.1557	0.12	0.5856	<0.001^a^
Android FFM (g)	−0.2286	0.02^a^	0.5647	<0.001^a^
Gynoid FFM (g)	−0.3223	<0.001^a^	0.5769	<0.001^a^
Residual mass (g)	−0.135	0.18	0.561	<0.001
Resting energy expenditure (kcal/day)	−0.369	<0.001^a^	‐	‐

BMI, body mass index; FFM, fat free mass; FM, fat mass.

a Indicate statistically significant parameters (*P *<* *0.05).

**Figure 6:**
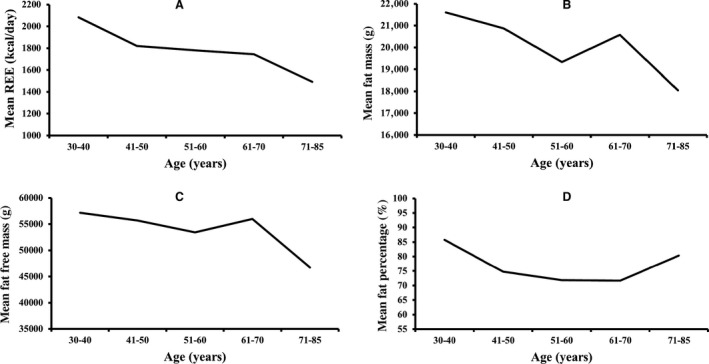
The variation of REE, FM, FFM, and Fat percentage with age. (A–D) The mean REE, mean FM, mean FFM, and mean fat percentage change across different age groups, respectively. REE, resting energy expenditure; FM, fat mass; FFM, fat free mass.

REE was more robustly correlated with total FFM (*r = *0.6245), than with total FM (*r = *0.4927). Furthermore, REE has a stronger relationship with trunk FFM (*r = *0.5856), lower limbs FFM (*r = *0.5738), upper limbs FFM (*r = *0.5537), RM (*r = *0.561), SF (*r = *0.417), and VF (*r = *0.380) than with trunk FM (*r = *0.4795), lower limbs FM (*r = *0.3960) and upper limbs FM (*r = *0.4434). The mean REE, mean FM, mean FFM, and mean fat percentage change across the different age groups are shown in Figure 6. Mean SF and VF relationship across age‐groups are depicted in Figure S1. Both FM and FFM decreased until about 55 years and increased in the succeeding 10 years, following which they decreased again. A similar trend was observed in SF and VF. Fat percentage gradually decreased until around 65 years and then increased thereafter. Overall, the analysis showed a significant inverse relationship of age with REE and FFM, indicating that advanced age was correlated with a decline in both REE and FFM. REE was also positively correlated with both regional and whole‐body FM and FFM, as well as fat distribution but correlated more strongly with FFM, which suggested that a significant decrease in FFM was accompanied by a decline in REE.

Linear regression analysis of age on REE as well as whole body and regional body composition variables indicated that age was significantly and independently related with FFM (variance=6.4%), but not with FM (variance = 2.9%)(See Table S1). Lower limbs FFM (variance = 10.7%), gynoid FFM (variance = 10.5%), upper limbs FFM (variance = 10.4%), RM (variance = 6.8%), and android FFM (variance = 5.2%) were also significantly associated with age. None of the FM variables, including total body and regional FM body composition, were significantly changed with age. Total body FM and FFM were included into the first multivariate regression model for age. The analysis demonstrated a significant and inverse association with FFM (*β = *−0.278, *P = *0.04) but revealed a non‐significant relationship with FM (*β = *0.031, *P = *0.82). In a more complex model adjusted for regional body composition variables, there was a considerably stronger and significant association of aging with trunk FFM (*β = *0.962, *P = *0.001) only whereas the other parameters entered into the model did not show any substantial association. The regression models are shown in Table S2.

When linear regression analysis was performed for the determinants of REE, (See Table S3) REE was significantly and more strongly associated with total body FFM which accounted for 39.0% of the variance compared to 24.3% of variability caused by total body FM. Trunk FFM (variance = 34.3%), gynoid FFM (variance = 33.3%), lower limbs FFM (variance = 32.9%), android FFM (variance = 31.9%), RM (31.7% variance), and trunk FM (variance = 23%) were also strong predictors of REE. Likewise, android FM (25.9% variance), upper limbs FM (variance = 19.7%), SF (variance = 17.4%), lower limbs FM (variance = 15.7%), gynoid FM (variance = 15.5%), VF (variance = 14.4%), and fat percentage (variance = 6.1%) significantly predicted the changes in REE. All the body composition variables being studied were found to significantly predict REE. The first multiple regression model for determining the predictors of REE, which included age, whole body FM and FFM, revealed that REE was significantly determined by age (*β =* −0.226, *P = *0.005) and FFM (*β = *0.489, *P *<* *0.001) with a variability of 44.4% in addition to no association with FM (*β = *0.111, *P = *0.30). After adjustment for regional body composition variables, only age remained a significant predictor of REE, suggesting that an age‐related reduction in REE separated from the changes in regional FM and FFM. Regression models are depicted in Table S4.

## Discussion

In this study, an advancing age strongly correlated with a decline in both FFM and REE. Nevertheless, an in‐depth analysis demonstrated that only trunk FFM accounted for age‐related changes in body composition. Age‐related decline in REE was strongly correlated to a decrease in total body and regional FM, FFM, SF, and VF, with a more robust correlation to FFM than FM, SF, and VF. However, only age significantly predicted REE after adjustments for regional body composition variables, independent of the changes in whole body and regional FM and FFM. These findings suggest that an age‐related decline in REE is not solely explained by changes in body composition that normally occurs with aging. It is noteworthy that advancing age was not correlated with FM and fat percentage but was associated with a decrease in body weight and BMI.

Geisler et al. (2016) and Bosy‐Westphal et al. (2003) reported that a lower REE was due to an age‐associated reduction in FFM as well as alterations in FFM composition. The decline of REE with advancing age could be explained by a lower metabolic activity. In addition to a decrease in both FFM and organ/tissue mass which contributed to FFM, the specific metabolic activity of distinct organs and tissues also accounted for a decrease of REE in older adults (Bosy‐Westphal et al. 2003; Geisler et al. 2016). Eighty percent of the variance in REE could be explained by the organs and tissues of FFM (Müller et al. 2013). In a cross‐sectional study which examined the relationship between REE and body composition, FFM was the main determinant of REE and only a small proportion of the remaining variance could be explained by FM (Nelson et al. 1992). These findings were in accordance with our results whereby FFM and FM accounted for 39% and 24.3% of variability in REE, respectively. However, since only age significantly predicted REE in multiple regression analysis, multicollinearity could have influenced the results.

In this study, an increase in FM was observed from 55 to 65 years, followed by a decrease in the 70–85 age group. Our results were consistent with Henche et al. (2008) who revealed that total body FM augmented up to 70 years of age and declined afterwards. Kyle et al. (2001) reported a subsequently lower appendicular and trunk FM after an initial rise until the age of 60–74 years old. Ding et al. (2007) showed that FM and fat percentage became gradually higher until 70–80 years old and stabilized from then on. Our results also demonstrated a rise in fat percentage after 70 years old. This occurrence is the consequence of a fast loss in lean mass in the elderly (Ding et al. 2007; Atlantis et al. 2008). In this study, the lower SF and VF present in the 70–85 age group is similar to the patterns observed in FM and was inconsistent with previous studies which described an increased SF and VF in older adults. Additionally, a decrease in body weight and BMI was also observed with age in the current study. To date, there are few studies which demonstrated a lower body weight, BMI and FM with advancing age in a Chinese population. Our findings were consistent with Kuk et al. (2009) who reported an age‐associated decrease of FM and weight in elderly Asians. In addition, Teh et al. (1996) revealed that a greater accumulation of fat in the abdominal region was accompanied by a decline in weight and BMI in elderly Chinese.

FM and weight differ across ethnicities and cultures as a result of lifestyle differences such as diet and physical activity. A total of 66.3% of Chinese individuals, aged between 35 and 74 years, were more engaged in light to moderate physical activities and men were reportedly more active than women (Muntner et al. 2005). In a study conducted by the Centers for Disease Control and Prevention, only 31.1% of American Asians males consumed fast food compared with 41.8%, 35.2%, and 39.0% of black, Hispanic and white men, respectively. They also reported that with advancing age, there is a decline in the proportion of adults consuming fast food (Centers for Disease Control and Prevention, 2018). Furthermore, Moore et al. (2009) described that Chinese subjects and those aged above 65 years old tend to have a healthier eating habit in contrast to other ethnicities and age groups. Therefore, the lower FM observed in our study could be explained by the likelihood that elderly Chinese are more engaged in physical activities and consumed less fast food.

With advancing age, a reduced appendicular FFM was observed in the study population. A prior longitudinal study which evaluated the changes of body composition showed that there was a significant reduction in total FFM and appendicular FFM in most elderly men but the total body FM was not significantly affected (Fantin et al. 2007). Another study also observed a substantial decrease in appendicular lean soft tissue mass in men compared to women after a follow‐up of 2 years (Visser et al. 2003). The decrease in appendicular FFM could be explained by the loss of skeletal muscle mass and bone density associated with aging (Novotny et al. 2015). REE was positively associated with all the parameters tested in our investigation. A decrease in REE was accompanied by a slight decrease in SF (*r = *0.417, R^2^ = 0.174) and VF (*r = *0.379, R^2^ = 0.144). SF and VF at L4 were taken into consideration as a measure for central adiposity. However, our findings are inconsistent with Jia et al. (2005) and Hunter et al. (2001) who showed that intraabdominal fat increase when there is a decline in age‐related REE in Chinese adults and white women, respectively.

It is widely accepted that physical activity maintains body composition by increasing FFM and lowering FM with aging (Manini 2010). Hunter et al. (2013) demonstrated that a combination of aerobic exercise and resistance training for 16 weeks was associated with a decrease in FM, an increase in FFM and no significant changes of REE in elderly women. Conversely, REE was increased in older adults after 26 weeks and 8 weeks of resistance training (Hunter et al. 2000) and high‐intensity aerobic exercises (Goran and Poehlman 1992), respectively. Van Pelt et al. (2001) explained a decline of REE in physically active elderly men by a decrease in weekly exercise training volume and daily energy intake. Since we did not assess physical activity level and energy intake, it is possible that these factors affected FM, SF, VF, FFM, and REE of the study population. In our study, the significant changes in trunk FFM with advancing age could therefore be present as a result of physical activity. The decreased REE in older adults could also be explained by lower organs/tissue activities or a reduction of the specific metabolic activity of distinct cells, or as a result of both (Manini 2010; Wang et al. 2010).

It is important to assess the relationship of age‐related decline in REE with FM, FFM, SF, and VF since all of these parameters have been implicated in physical disabilities and diseases as well as with an increased mortality and morbidity risk. A study which examined the relationship of body composition and mortality revealed that extremes in FM and reduced lean mass were associated with higher mortality risk in elderly men (Toss et al. 2012). Han et al. (2010) demonstrated that a lower lean body mass and lean BMI were linked with higher mortality in elderly Asians. However, they found no relationship between FM and fat components with mortality. A previous investigation showed that a decline in appendicular FFM was related to increased disability (Fantin et al. 2008). Further research should explore the age‐related decrease in REE on disability, mortality, and morbidity.

In summary, our study on Chinese Han males was in agreement with previous investigations indicating that a lower REE was associated with advancing age. Nevertheless, aging was independently associated with a decline in REE, regardless of age‐associated changes in body composition, thereby suggesting that REE is not solely explained by body composition changes. The alterations in body composition observed with aging was explained by trunk FFM changes. Additionally, FM increased up until 55–65 years followed by a decline thereafter. The elderly Chinese men also had lower VF and SF. These findings could be explained by cultural and ethnical disparities, whereby diet and physical activities played a key role. Further research should be performed to understand why REE, FM, and FFM declined with aging in the Chinese population and the impact of these factors on overall health.

## Limitations

One limitation of this study is the relatively small sample size. The participants were healthy individuals and those older than 65 years old might not be representative of the geriatric population. The protocol of the study was cross‐sectional and thus, the observed age‐related changes in body composition and fat distribution could be interpreted as the variations in consecutive age groups. Since a longitudinal study was not performed, we could not assess the disability, mortality, and morbidity associated with aging and age‐associated decline in REE. The physical activity level and energy intake of the subjects were not assessed and could potentially affect the results. Medications and nutritional supplements were not taken into consideration and could also influence our findings.

## Ethical Standards

All research procedures were in accordance with the ethical principles set by the World Medical Association Declaration of Helsinki (World Medical Association, 2013) and the research review board of The First Affiliated Hospital of Nanjing Medical University approved the current study. All participants provided informed consent prior to inclusion in the study.

## Conflicts of Interest

The authors have no conflict of interest to declare.

## Supporting information




**Table S1.** Linear regression analysis of age with body composition in Chinese Han men.
**Table S2.** Multivariate regression models for estimating the association of different variables with age.
**Table S3.** Linear regression analysis for identifying the best predictors of REE with respect to whole body and regional body composition in Chinese Han men.
**Table S4.** Multiple regression models for estimating the association of different variables with REE.
**Figure S1.** The variation of SF and VF with age. (A) and (B) shows the SV and VF change across different age groups. SF, Subcutaneous Fat; VF, Visceral Fat.
