# Supplementary material for: Age‐associated changes of resting energy expenditure, body composition and fat distribution in Chinese Han males
Source: Physiol Rep. 2018 Dec 8;6(23):e13940. doi: 10.14814/phy2.13940 (PMC6286433; doi:10.14814/phy2.13940)
Supplement: Supplementary file 1 — Table S1. Linear regression analysis of age with body composition in Chinese Han men. Table S2. Multivariate regression models for estimating the association of different variables with age. Table S3. Linear regression analysis for identifying the best predictors of REE with respect to whole body and regional body composition in Chinese Han men. Table S4. Multiple regression models for estimating the association of different variables with REE. Figure S1. The variation of SF and VF with age. (A) and (B) shows the SV and VF change across different age groups. SF, Subcutaneous Fat; VF, Visceral Fat. [file PHY2-6-e13940-s001.docx]

**Supplementary Materials**

**Supplementary Figure 1**

**Supplementary Table 1.** Linear Regression Analysis of age with body composition in Chinese Han men.

| Variables | *R* | *R^2^* | *p-*value | 95% CI | | Standard Error |
| --- | --- | --- | --- | --- | --- | --- |
|  |  |  |  | Lower bound | Upper bound |  |
| FM | 0.169 | 0.029 | 0.09 | 53.392 | 70.892 | 4.410 |
| FFM | 0.254 | 0.064 | 0.01* | 59.925 | 90.861 | 7.795 |
| Fat percentage (%) | 0.010 | 0.000 | 0.923 | 49.174 | 61.370 | 3.073 |
| Upper Limbs FM | 0.193 | 0.037 | 0.052 | 54.363 | 68.735 | 3.622 |
| Lower Limbs FM | 0.115 | 0.013 | 0.25 | 51.105 | 68.052 | 4.271 |
| Gynoid FM | 0.139 | 0.019 | 0.16 | 51.954 | 69.710 | 4.474 |
| Trunk FM | 0.163 | 0.027 | 0.10 | 53.334 | 68.554 | 3.835 |
| Android FM | 0.181 | 0.033 | 0.07 | 54.114 | 67.573 | 3.391 |
| Upper Limbs FFM | 0.322 | 0.104 | 0.001* | 63.345 | 87.088 | 5.983 |
| Lower Limbs FFM | 0.328 | 0.107 | 0.001* | 65.185 | 93.663 | 7.176 |
| Gynoid FFM | 0.324 | 0.105 | 0.001* | 65.269 | 93.077 | 7.007 |
| Trunk FFM | 0.156 | 0.024 | 0.12 | 52.063 | 80.572 | 7.184 |
| Android FFM | 0.229 | 0.052 | 0.02* | 57.110 | 80.531 | 5.902 |
| SF | 0.075 | 0.006 | 0.45 | 50.419 | 63.962 | 3.413 |
| VF | 0.090 | 0.008 | 0.37 | 51.241 | 63.340 | 3.049 |
| Residual Mass | 0.260 | 0.068 | 0.009* | 49.779 | 79.706 | 7.541 |

*R* represents the correlation between age and the respective body composition. *R^2^* represents the proportion of variance. A *p-*value of *<* 0.05 indicates significant association, denoted by *. FM: Fat Mass; FFM: Fat Free Mass. CI denotes the confidence interval.

**Supplementary Table 2.** Multivariate regression models for estimating the association of different variables with age.

| Variables | *R*^2^ | Beta | *P*-value |
| --- | --- | --- | --- |
| 1. Constant   FM  FFM | 0.066  -  - | -  0.031  -0.278 | 0.04  0.82  0.04* |
| 1. Constant   ULFM  LLFM  GFM  TFM  AFM  ULFFM  LLFFM  GFFM  TFFM  AFFM  SF  VF | 0.269  -  -  -  -  -  -  -  -  -  -  -  - | -  -0.077  0.344  -0.197  -0.030  -0.336  -0.385  -0.248  -0.359  0.926  -0.191  0.207  0.052 | <0.001  0.70  0.20  0.56  0.93  0.40  0.08  0.23  0.23  0.001*  0.42  0.235  0.71 |

*R^2^* represents the proportion of variance. A *p-*value of *<* 0.05 indicates significant association. FM: Fat Mass; FFM: Fat Free Mass; ULFM: Upper Limbs Fat Mass; LLFM: Lower Limbs Fat Mass; GFM: Gynoid Fat Mass; TFM: Trunk Fat Mass; AFM: Android Fat Mass; ULFFM: Upper Limbs Fat Free Mass; LLFFM: Lower Limbs Fat Free Mass; GFFM: Gynoid Fat Free Mass; TFFM: Trunk Fat Free Mass; AFFM: Android Fat Free Mass; SF: Subcutaneous Fat; VF: Visceral Fat.

**Supplementary Table 3.** Linear Regression Analysis for identifying the best predictors of REE with respect to whole body and regional body composition in Chinese Han men.

| Variables | *R* | *R^2^* | *p*-value | 95% CI | | Standard Error |
| --- | --- | --- | --- | --- | --- | --- |
|  |  |  |  | Lower bound | Upper bound |  |
| Age | 0.369 | 0.136 | <0.001 | 2064.096 | 2648.632 | 147.296 |
| FM | 0.493 | 0.243 | <0.001 | 965.257 | 1400.624 | 10.783 |
| FFM | 0.625 | 0.390 | <0.001 | 29.431 | 733.785 | 177.489 |
| Fat percentage (%) | 0.246 | 0.061 | 0.01 | 1417.323 | 1750.607 | 83.984 |
| Upper Limbs FM | 0.443 | 0.197 | <0.001 | 1158.659 | 1528.232 | 93.128 |
| Lower Limbs FM | 0.396 | 0.157 | <0.001 | 1100.445 | 1542.009 | 111.269 |
| Gynoid FM | 0.394 | 0.155 | <0.001 | 1066.581 | 1530.943 | 117.014 |
| Trunk FM | 0.480 | 0.230 | <0.001 | 1089.665 | 1471.205 | 96.144 |
| Android FM | 0.509 | 0.259 | <0.001 | 1148.479 | 1480.513 | 83.669 |
| Upper Limbs FFM | 0.554 | 0.307 | <0.001 | 518.509 | 1107.682 | 148.465 |
| Lower Limbs FFM | 0.574 | 0.329 | <0.001 | 225.991 | 921.795 | 175.335 |
| Gynoid FFM | 0.577 | 0.333 | <0.001 | 250.980 | 928.138 | 170.636 |
| Trunk FFM | 0.586 | 0.343 | <0.001 | 270.069 | 929.586 | 166.191 |
| Android FFM | 0.565 | 0.319 | <0.001 | 555.137 | 1114.973 | 141.072 |
| SF | 0.417 | 0.174 | <0.001 | 1228.339 | 1576.164 | 87.648 |
| VF | 0.379 | 0.144 | <0.001 | 1318.715 | 1635.251 | 79.764 |
| Residual Mass | 0.624 | 0.390 | <0.001 | 241.841 | 944.980 | 177.183 |

*R* represents the correlation between REE and respective body composition. *R^2^* represents the proportion of variance. A *p-*value of *<* 0.05 indicates significant association. FM: Fat Mass; FFM: Fat Free Mass. CI denotes the confidence interval.

**Supplementary Table 4.** Multiple Regression models for estimating the association of different variables with REE.

| Variables | *R*^2^ | Beta | *P*-value |
| --- | --- | --- | --- |
| 1. Constant   Age  FM  FFM | 0.444  -  -  - | -  -0.226  0.111  0.489 | <0.001  0.005*  0.30  <0.001* |
| 1. Constant   Age  ULFM  LLFM  GFM  TFM  AFM  ULFFM  LLFFM  GFFM  TFFM  AFFM  SF  VF  RM | 0.477  -  -  -  -  -  -  -  -  -  -  -  -  -  - | -  -0.254  -0.144  0.362  -0.437  0.187  0.086  0.238  0.129  -0.270  0.317  0.120  0.070  0.018  -0.164 | <0.001  0.006*  0.40  0.12  0.13  0.55  0.80  0.21  0.46  0.30  0.18  0.56  0.60  0.88  0.87 |

*R^2^* represents the proportion of variance. A *p-*value of *<* 0.05 indicates significant association. FM: Fat Mass; FFM: Fat Free Mass; ULFM: Upper Limbs Fat Mass; LLFM: Lower Limbs Fat Mass; GFM: Gynoid Fat Mass; TFM: Trunk Fat Mass; AFM: Android Fat Mass; ULFFM: Upper Limbs Fat Free Mass; LLFFM: Lower Limbs Fat Free Mass; GFFM: Gynoid Fat Free Mass; TFFM: Trunk Fat Free Mass; AFFM: Android Fat Free Mass; SF: Subcutaneous Fat; VF: Visceral Fat.

**Figure Legends**

**Supplementary Figure 1.** The variation of SF and VF with Age.

(A) and (B) shows the SV and VF change across different age groups. SF: Subcutaneous Fat; VF: Visceral Fat.

**Table Legends**

**Supplementary Table 1.** Linear Regression Analysis of age with body composition in Chinese Han men.

**Supplementary Table 2.** Multivariate regression models for estimating the association of different variables with age.

**Supplementary Table 3.** Linear Regression Analysis for identifying the best predictors of REE with respect to whole body and regional body composition in Chinese Han men.

**Supplementary Table 4.** Multiple Regression models for estimating the association of different variables with REE.
